# Insights on interferon-independent induction of interferon-stimulated genes shaping the lung's response in early SARS-CoV-2 infection

**DOI:** 10.1016/j.heliyon.2023.e22997

**Published:** 2023-11-28

**Authors:** Sung-Dong Cho, Haeun Shin, Sujin Kim, Hyun Jik Kim

**Affiliations:** a Graduate School of Medical Science and Engineering, Korea Advanced Institute of Science and Technology, Daejeon, South Korea; b Department of Otorhinolaryngology, Seoul National University College of Medicine, Seoul, South Korea; c Sensory Organ Research Institute, Seoul National University Medical Research Center, Seoul, Korea

**Keywords:** SARS-CoV-2, Interferon-stimulated genes, Innate immune responses, Interferon-independent pathway

## Abstract

While mRNA vaccine efficacy against the 2019 coronavirus disease (COVID-19) outbreak remains high, research on antiviral innate immune responses in the early stages of infection is essential to develop strategies to prevent the dissemination of SARS-CoV-2. In this study, we investigated the induction of both interferon (IFN)-stimulated genes (ISGs) and IFN-independently upregulated ISGs following SARS-CoV-2 infection in Syrian golden hamsters. The viral titers were highest at 3 days post-infection (dpi). Over time, the viral titer gradually decreased while ISGs such as *Mx1*, *Ifit2*, *Ifit3*, *Ifi44*, and *Rsad2* were markedly induced in the lung. The transcription of ISGs significantly increased from 2 dpi, and SARS-CoV-2-induced ISGs were maintained in the hamster lung until 7 dpi. The transcription of *Ifnb* and *Ifng* was minimally elevated, while *Ifnl*_*2/3*_ was significantly induced in the lung at 5 days after SARS-CoV-2 infection. RNA sequencing results also showed that at 3 dpi, SARS-CoV-2 initiated the activation of ISGs, with lesser increases of *Ifnl*_*2*_ and *Ifnl*_*3*_ transcription. In addition, *Ddx58* and *cGAS*, which encode factors for virus sensing, *Stat1*, *Stat2,* and *IFN regulatory factor 7* and 9 mRNA levels were also induced at the initial stage of infection. Our data demonstrate that ISGs might be upregulated in the lung in response to SARS-CoV-2 during the early stages of infection, and the rapid induction of ISGs was not associated with the activation of IFNs. Elucidation of IFN-independent induction of ISGs could further our understanding of alternative defense mechanisms employed by the lungs against SARS-CoV-2 and provide more effective antiviral strategies for patients with severe COVID-19.

## Highlights

• Interferon-stimulated genes (ISGs) might be upregulated in the lungs of hamsters in response to SARS-CoV-2.
• The rapid induction of ISGs served a critical role in facilitating innate immune responses.
• Early-stage ISG induction in SARS-CoV-2-infected hamster lungs was not associated with interferon (IFN) activation.
• IFN-independent responses against SARS-CoV-2 limit the viral replication in the respiratory tract with lower IFN production.

## 1 Introduction

The developed vaccines have curtailed the dissemination of severe acute respiratory syndrome coronavirus 2 (SARS-CoV-2), and vaccine-activated adaptive immune responses decreased the severity of coronavirus disease 2019 (COVID-19), even as new variants have emerged [1,2]. As a defense mechanism against SARS-CoV-2 infection in the respiratory tract mucosa, both innate immune response and potent adaptive immunity play an important role. However, the efficacy of heightened innate immune responses following SARS-CoV-2 infection has not yet been clearly identified. Virus-specific innate immunity has been thought to be exclusively initiated by type I and III interferons (IFNs) in the respiratory tract and facilitates intracellular antiviral signaling through transcription of IFN-stimulated genes (ISGs) [3,4]. In a coordinated fashion, the immune systems with type I and type III IFNs are necessary to restrict viral replication, and successful clearance of viral pathogens through induction of ISGs is required for effective control of acute viral lung infection.

Recent data have shown that SARS-CoV-2 has developed strategies to counteract IFNs, and SARS-CoV-2 efficiently antagonizes IFN effects that fail to suppress viral replication at the initial stage of infection [5]. In individuals with severe disease, SARS-CoV-2 enhances the production of inflammatory mediators while suppressing antiviral IFN responses [6]. The production of ISGs following SARS-CoV-2 infection appears to be more complex than other respiratory viruses, and our knowledge of the contributions of IFNs and ISGs to restrict the spread of SARS-CoV-2 along the respiratory mucosa is limited. Thus, investigating the crosstalk between IFNs and ISGs in the lung's response to SARS-CoV-2 can offer insights into protective immunity against the virus and enhance immunity during the early stages of infection.

Here, we demonstrated that the ISGs in the lungs of hamsters infected with SARS-CoV-2 were rapidly induced after the onset of viral replication, and the rapid upregulation of ISGs in response to SARS-CoV-2 appeared to be activated before significant induction of IFNs, which were not required for the increase of ISGs at an early stage of lung infection. Our work reveals insight into the IFN-independent transcriptional regulation of ISGs in the lung against SARS-CoV-2, which might be involved in a defense mechanism to limit the viral replication in COVID-19 patients with lower IFN production.

## 2 Materials and methods

### 2.1 Viruses and reagents

A SARS-CoV-2 strain (BetaCov/Korea/SNU01/2020) was used to induce acute respiratory viral infection. Virus stocks were cultured in Vero cells (ATCC® CCL-81™) with viral growth medium according to standard procedures [7]. The viral stock was inoculated into the monolayers of Vero cells and was cultured at 37 °C, 5 % CO_2_ for 48 h. The cells were centrifuged at 5000 rpm for 30 min, then the supernatants were obtained. The virus was titrated with a tissue culture infectious dose (TCID) assay. The virus stock was stored at −80 °C.

### 2.2 Hamsters and SARS-CoV-2 infection

The Institutional Review Board (IRB) of Seoul National University College of Medicine approved the protocol for this study (IRB #2020059). Experiments with Syrian golden hamsters were conducted according to guidelines approved by the IACUC of the Seoul National University College of Medicine (IACUC #20-0219-S1A0).

Male Syrian golden hamsters, aged 7 weeks, obtained from Orientbio in Seoul, Korea, were used as the SARS-CoV-2 infection model. These hamsters were intranasally inoculated with SARS-CoV-2 at a 5.0 x104 TCID50/hamster dose in 30 μL of phosphate-buffered saline (PBS). Bronchoalveolar lavage (BAL) fluid was collected with 1000 μL of 0.5 mM ethylene diamine tetraacetic acid in PBS. We conducted real-time polymerase chain reaction (PCR), bulk RNA-Seq, and immunohistochemistry using these samples.

### 2.3 Real-time PCR

Real-time PCR was conducted following the methods previously described [8]. Primers for hamster *Ifnb*, *Ifnl*_*2/3*_, *Mx1*, *Ifit2*, *Ifit3*, and *Rsad2* were purchased from Applied Biosystems (Foster City, CA, USA). Target mRNA levels were quantified using target-specific primer and probe sets for *S gene*, *Ifnb*, *Ifnl*_*2/3*_, *Mx1*, *Ifit2*, *Ifit3*, and *Rsad2*. All PCR assays were quantitative, and plasmids containing the target gene sequences were used as standards. All real-time PCR data except those of the *S gene* were normalized to the level of glyceraldehyde phosphate dehydrogenase mRNA (1 × 10^6^ copies) to correct for variations between samples.

### 2.4 Immunohistochemistry and histologic analysis

Immunohistochemistry was conducted following the methods previously described [8].

### 2.5 Bulk RNA sequencing

#### 2.5.1 RNA isolation

After the isolation of total RNA, RNA quality was assessed with an Agilent 2100 bioanalyzer using the RNA 6000 Nano Chip (Agilent Technologies, Amstelveen, Netherlands). RNA quantification was performed using an ND-2000 Spectrophotometer (Thermo Inc., DE, USA).

#### 2.5.2 Library preparation and sequencing

For control and test RNAs, library construction was performed using the QuantSeq 3′ mRNA-Seq Library Prep Kit (Lexogen, Inc., Austria) according to the manufacturer's instructions. In brief, total RNA was prepared, and an oligo-dT primer containing an Illumina-compatible sequence at its 5′ end was hybridized to the RNA; reverse transcription was then performed. After degradation of the RNA template, second strand synthesis was initiated by a random primer containing an Illumina-compatible linker sequence at its 5′ end. The double-stranded library was purified using magnetic beads to remove all reaction components. The library was amplified to add the complete adapter sequences required for cluster generation. The finished library was purified from PCR components. High-throughput sequencing was performed as single-end 75 sequencing using a NextSeq 500 (Illumina, Inc., USA).

#### 2.5.3 Data analysis

QuantSeq 3’ mRNA-Seq reads were aligned using Bowtie2 [9]. Bowtie2 indices were either generated from a genome assembly sequence or the representative transcript sequences for aligning to the genome and transcriptome. The alignment file was used for assembling transcripts, estimating their abundance, and detecting the differential expression of genes. Differentially expressed genes were determined based on counts from unique and multiple alignments using coverage in Bedtools [10]. The Read Count (RC) data were processed based on the TMM + CPM normalization method using EdgeR within R (R Development Core Team, 2020) using Bioconductor [11]. Gene classification was based on searches performed in DAVID (http://david.abcc.ncifcrf.gov/) and Medline databases (http://www.ncbi.nlm.nih.gov/). Data mining and graphic visualization were performed using ExDEGA (Ebiogen Inc., Korea). The cutoff criteria were defined as adjusted *p* < 0.05, fold change ≥ or <2.0, and normalized data (log2) ≥ or < 4.0. The original bulk RNA-Seq data for hamster models with SARS-CoV-2 infection are available at https://www.ncbi.nlm.nih.gov/geo/query/acc.cgi?acc=GSE204696.

### 2.6 Statistical analyses

Data from *in vivo* results of real-time PCR were presented as mean ± SD values from three individual hamsters with three independent experiments and differences between treatment groups were evaluated by repeated measure two-way analysis of variance (ANOVA). Two-sample t-tests were used to analyze the bulk RNA-Seq data. GraphPad Prism (version 9; GraphPad Software, La Jolla, CA, USA) was used for statistical analyses, and a *p*-value <0.05 indicated statistical significance.

## 3 **Results**

**Fig. 1:**
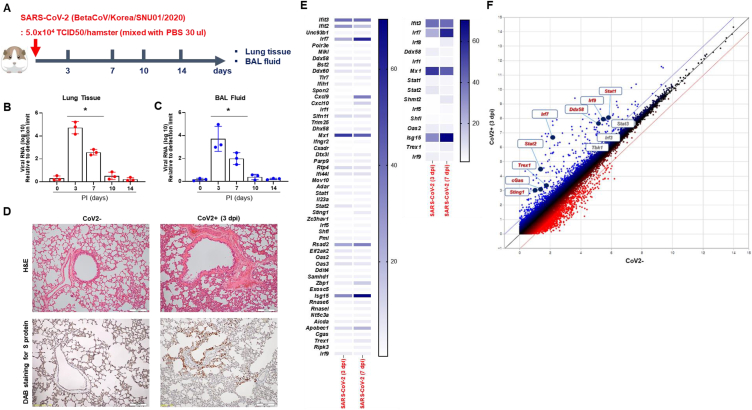
IFN- and ISG-related innate immune responses in the lung of CoV2+ hamsters. A. Scheme of the hamster model and the design of experiment for SARS-CoV-2 infection. Syrian golden hamsters were infected with SARS-CoV-2 (BetaCoV/Korea/SNU01/2020, 2.5 × 10^5^ TCID50/hamster) at the indicated time points (N = 3 at each indicated time point). B, C. *Spike* RNA levels in lung tissue (B) and BAL fluid (C) were determined at 0, 3, 7, 10, and 14 dpi. D. H&E-stained micrographs were generated from lung sections of hamsters at indicated time points of SARS-CoV-2 infection (scale bar, 100 μM). Immunohistochemical analysis of spike protein using DAB chromogen was performed in lung sections from hamsters prior to and following SARS-CoV-2 infection. Micrographs shown are representative of lung sections from three hamsters (original magnification x200). E. Differentially expressed genes (DEGs) were identified to further characterize the transcriptional changes in response to SARS-CoV-2. F. Scattered plots indicate DEGs correlated with “virus recognition” and “transcription factors of IFNs or ISGs.” Real-time PCR results were analyzed by the Mann–Whitney *U* test. Data are presented as mean ± SD from three independent experiments. *p < 0.05 vs. non-infected hamsters.

SARS-CoV-2 was intranasally delivered into Syrian golden hamsters (referred to as CoV2+ hamsters) (Fig. 1A). The viral RNA (*S* gene) and lung histologic findings were examined using lung tissue and BAL fluid at 0, 3, 7, 10, and 14 dpi. Viral RNA titers in CoV2+ hamster lungs were significantly elevated, peaking at 3 dpi (5.1 × 10^5^); and gradually decreasing from 7 dpi (Fig. 1B). Viral RNA was not detected at 10 and 14 dpi, nor in the BAL fluid of CoV2+ hamsters (Fig. 1C). In CoV2+ hamsters, the examination of lung sections stained with H&E showed a notable infiltration of inflammatory cells around the bronchioles at 3 dpi. Immunohistochemistry analysis revealed the presence of the SARS-CoV-2 spike protein, with a significant increase in cell density observed in bronchial epithelial cells at 3 dpi. (Fig. 1D).

The transcriptional alterations were further characterized using bulk RNA-Seq, and we analyzed differentially expressed genes (DEGs) from lung lysates after 3 and 7 days post-infection. Comparison of lung lysates of CoV2+ and CoV2- hamsters revealed 32,315 DEGs (cutoff criteria of over 4 (log2) fold change) in the lungs. The associated gene ontology (GO) term was examined by DAVID analysis, and 55 relevant DEGs were observed in the “defense to virus” category at 3 or 7 dpi (Fig. 1E). The transcription of *Ifit3*, *Ifit2*, *Irf7*, *Slfn11*, *Mx1*, *Rtp4*, *Ifi44l*, *Stat2*, *Rsad2*, *Apobec1*, *Usp18*, and *Isg15* was significantly elevated from 3 dpi. The associated GO term “response to type I or III interferons” was also examined, and 15 relevant DEGs were observed at 3 or 7 dpi (Fig. 1E). Scattered data plot showed that the transcription factors of ISGs (*Stat1*, *Stat2*, *Irf7*, *Irf9*) and the genes related to virus recognition (*cGAS*, *Trex1*, *Ddx58*) showed significantly higher levels at 3 dpi (Fig. 1F).

**Fig. 2:**
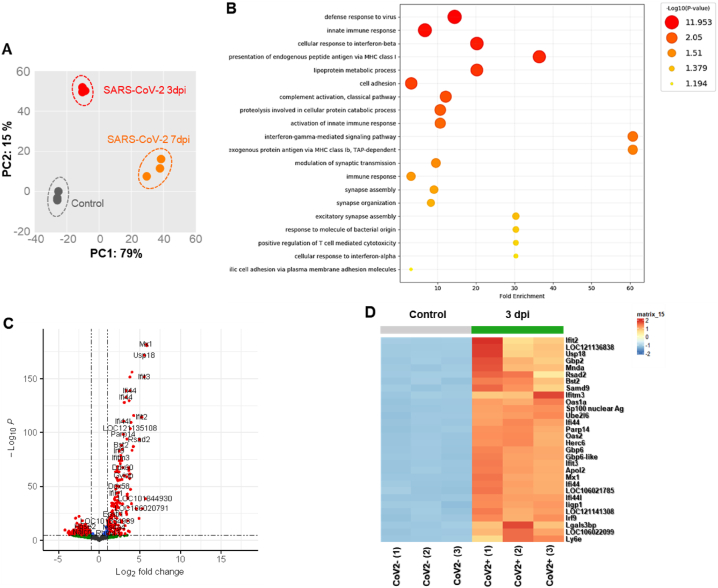

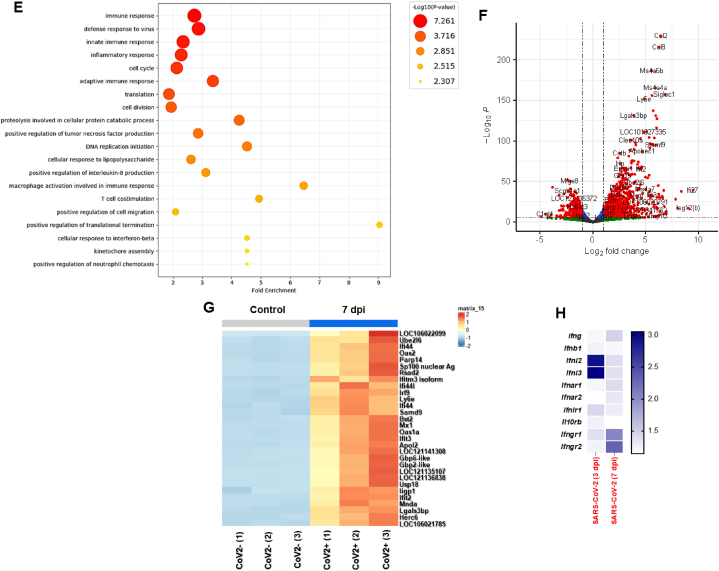
**Transcriptional alterations in the lung of CoV2+ hamsters.** Syrian golden hamsters (N = 15) were infected with SARS-CoV-2 (BetaCoV/Korea/SNU01/2020, 2.5 × 10^5^ TCID50/hamster), and the lung lysate was prepared at 3 and 7 dpi (N = 3 at each indicated time point). A. Principal component analysis (PCA) was performed to reveal the similarities and dissimilarities of 3 groups (0, 3, and 7 dpi) regarding variations in DEGs. B. The GO terms in the lung of SARS-CoV-2 infection at 3 dpi. C. Volcano plot data were examined, and 490 DEGs were observed in the lungs of SARS-CoV-2-infected hamsters, especially at 3 dpi. D. The heatmap shows the top 30 significant DEGs in the lungs of hamsters following SARS-CoV-2 infection at 3 dpi. E. The GO terms in the lung of SARS-CoV-2 infection at 7 dpi. F. Volcano plot data were examined, and 2582 DEGs were observed in the lungs of SARS-CoV-2-infected hamsters, especially at 7 dpi. G. The heatmap shows the top 30 significant DEGs in the lungs of hamsters following SARS-CoV-2 infection at 7 dpi. H. The relative expression of genes associated with IFNs and IFN-specific receptors was examined in the lungs of CoV2+ hamsters, especially at 3 and 7 dpi.

To gain more insight into transcriptional phenotypes linked to the expression of ISGs and IFNs, we additionally investigated the RNA-seq data using the tMod R package. We first performed a principal component analysis (PCA) to reveal the similarities and dissimilarities of three groups (0 dpi, 3 dpi, and 7 dpi) regarding variations in DEGs. PCA plot according to the DEGs of CoV2+ hamster lungs were separated into the first principal component at 3 dpi and 7 dpi (PC1, 53 % proportion) (Fig. 2A).

There were 490 DEGs at 3 dpi CoV2+ hamsters compared with CoV2- hamsters. GO enrichment analysis of biological processes revealed that the DEGs were mainly enriched in “defense response to virus”, “innate immune response”, and “cellular response to interferon beta” (Fig. 2B). The bulk RNA-Seq data revealed that transcription of *Mx1*, *Ifi44*, *Ifit2*, *Ifit3*, *Ifitm3*, and *Rsad2* was significantly upregulated at 3 dpi (Fig. 2C). Notably, the level of *Ifnl3* was low in the lung of hamsters in response to SARS-CoV-2, and the other core signatures of IFNs such as *Ifna*, *Ifnb*, and *Ifnl*_*2*_ were not induced at 3 dpi even if ISGs were induced following SARS-CoV-2 infection. The top 30 DEGs which were induced at 3 dpi included signature ISGs such as *Ifit2, Iigp1 (IFN-inducible GTPase1), Tgtp2 (T cell-specific GTPase2), Ifitm3,* and *Ifi44* (Fig. 2D).

There were 2582 DEGs at 7 dpi CoV2+ hamster lungs compared with CoV2- hamsters, and the GO analysis of biological processes showed that the DEGs were mainly enriched in “immune response”, “defense response to virus”, “innate immune response”, and “inflammatory response” (Fig. 2E). The bulk RNA-Seq data revealed significantly higher gene expression of *Ms4a6b*, *Ccl8*, *Ccl2*, *Ifit3*, and *Rsad2* at 7 dpi. Surprisingly, transcription of *IFNs* was not elevated at 7 dpi (Fig. 2F). The top 30 most significant DEGs related to ISGs were characterized in the 7 dpi CoV2+ hamsters. *Mx1*, *IfitIfi44, Ifit3, Rsad2,* and *Gvinp1 (GTPase, very large IFN-inducible pseudogene1)* were significant in the cluster of 7 dpi CoV2+ hamsters (Fig. 2G). We next determined the transcription levels of *Ifna*, *Ifnb1*, *Ifnl2*, *Ifnl3*, and *Ifng* at 0, 3, and 7 dpi, including the receptors of IFNs. The bulk RNA-Seq results revealed slightly elevated transcription of *Ifnl2* (2.92-fold over CoV2-) and *Ifnl3* (3.051-fold over CoV2-) at 3 dpi. No IFNs were induced at 7 dpi (Fig. 2H).

**Fig. 3:**
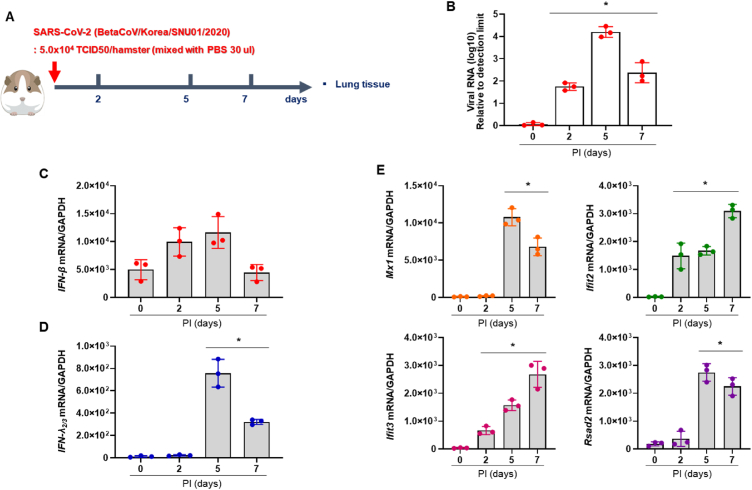
**Differential gene expression of IFNs and ISGs at early onset of SARS-CoV-2 infectio**n A. Schematic of the hamster model and experimental design for SARS-CoV-2 infection. Syrian golden hamsters (N = 12) were infected with SARS-CoV-2, and the lung lysate was prepared at 0, 2, 5, and 7 dpi. B. Viral *S* gene RNA in lung tissue was determined at 0, 3, and 7 dpi. C, D. The mRNA levels of *Ifnb1* (C) and *Ifnl*_*2/3*_ (D) were determined in the lungs of SARS-CoV-2-infected hamsters at 0, 2, 5, and 7 dpi. E. The mRNA levels of *Mx1, Ifit2, Ifit3, and Rsad2* were determined in the lungs of SARS-CoV-2-infected hamsters at 0, 2, 5, and 7 dpi. The real-time PCR results were analyzed by the Mann–Whitney *U* test and are presented as mean ± SD from three independent experiments. *p < 0.05 vs. non-infected hamsters.

To examine the distinct patterns of ISGs and IFNs in the hamster lungs at earlier stages of SARS-CoV-2 infection, we assessed viral RNA levels and measured mRNA expression of IFNs and signature ISGs using cell lysates from CoV2+ hamsters at 2, 5, and 7 dpi (Fig. 3A). The real-time PCR results indicated a significant rise in *S* RNA levels from 2 dpi, reaching its peak at 5 dpi (4.3 × 10^4^), followed by a gradual decline (Fig. 3B). Our preliminary data showed that viral RNA level was not elevated at 1 dpi (unpublished data). The *Ifnb*_*1*_ mRNA level was not induced at 2 dpi and was minimally increased at 5 dpi (1.34-fold over CoV2-) in CoV2+ hamster lungs (Fig. 3C). *Ifnl*_*2/3*_ mRNA levels were upregulated in the lung of SARS-CoV-2-infected hamsters at 5 dpi but decreased abruptly from 7 dpi; none was detected at 2 dpi (Fig. 3D). In contrast, transcription of *Ifit2* and *Ifit3* was significantly elevated from 2 dpi, and the highest levels were observed at 7 dpi (Fig. 3E). The gene expression of *Mx1* and *Rsad2* was upregulated at 5 dpi, and then the induced gene expressions were maintained until 7 dpi. Neither ISG nor IFN was induced in CoV2+ hamster lungs at 1 dpi (unpublished data). We speculated that these ISGs were induced at the early stage of infection in the lung in concert with the onset of SARS-CoV-2 replication. The rapid induction of ISGs in response to SARS-CoV-2 might be regulated by distinctive signals irrespective of IFN activities.

## 4 Discussion

Here, we showed that upregulation of ISGs in the lung occurred rapidly after the onset of SARS-CoV-2 infection, and IFNs were not required for induction of ISGs at early SARS-CoV-2 infection. Our study highlights the possibility of IFN-independent activation of ISGs and proposes an alternative antiviral landscape to defend against SARS-CoV-2-dampened antiviral IFN responses in COVID-19 patients.

While neutralizing antibodies and vaccines designed to target the spike protein of SARS-CoV-2 have offered protection to numerous patients [12,13], the development of a therapeutic agent capable of rapidly inhibiting the spread of SARS-CoV-2 during the early stages of infection is still limited. The use of IFNs provides a superior advantage as a host-targeted immune inducer that is efficacious for treating SARS-CoV-2 infection [14]. To this end, understanding the pattern of antiviral genes and ISGs activated by IFNs in the respiratory tract after SARS-CoV-2 infection is essential.

Our study revealed that ISGs such as *Mx1*, *Ifit2*, *Ifit3*, *Apobec1*, *Ifi44*, and *Rsad2* exhibited a more rapid induction in the lungs of SARS-CoV-2 infected hamsters, starting from 2 or 3 dpi. These genes may play a preferential role in mediating early-stage antiviral responses. Moreover, the elevated transcription of ISGs was maintained until 7 dpi following SARS-CoV-2 infection. Remarkably, although there was an increased transcription of ISGs in the lung in response to SARS-CoV-2 infection at 2dpi, the induction of *IFN-λs* did not occur until 5 dpi, and the mRNA levels of either *IFN-β* or *IFN-γ* in the lungs of CoV2+ hamsters showed only minimal elevation. Our estimation suggests that the upregulation of ISGs during early stages following SARS-CoV-2 infection serves as the primary antiviral defense mechanism in the lung and that IFNs are not necessary for the rapid induction of ISGs in the initial stage of SARS-CoV-2 replication.

Severely ill COVID-19 patients have exhibited the absence of circulating type I IFNs, which might correlate with the severity of COVID-19 and SARS-CoV-2 has evolved effective mechanisms to suppress the production of host IFNs in the respiratory tract [5]. There was also a suggestion that mutations in other genes related to type I IFN might be present in other patients experiencing life-threatening COVID-19 pneumonia (Our data also revealed that the transcription of core IFNs was low in the lung of CoV2+ hamsters and began to increase slightly from 3 or 5 dpi. However, a significantly higher level of viral RNA of SARS-CoV-2 from 2 dpi, and was gradually attenuated from 7 dpi in the lung of CoV2+ hamsters. The current findings suggest the possibility of an alternative pathway that induces ISG transcription in the lung for suppression of SARS-CoV-2 irrespective of IFN induction.

Infection with enveloped viruses initiates an IFN-independent pathway that directly produces a subset of ISGs [15]. In addition, a pathway was identified in the innate immune system involving cGAS and STING that mediates the signaling for IFN-independent activation of ISGs [16]. In fact, directly activating the antiviral genes is necessary for “nonprofessional” IFN-producing cells to mount an effective defense against viruses that have developed strategies to interfere with the IFN response. The rapid induction of IFN-independent ISGs performs a crucial functional role in suppressing viral replication before the more robust and sustained activation by IFNs begins [17].

Our data showed that ISGs such as *Mx1*, *Ifit2*, *Ifit3*, *Apobec1*, *Ifi44*, and *Rsad2* were significantly induced from 2 dpi following infection by the enveloped virus SARS-CoV-2 with lesser induction of IFNs, and the elevated transcription of ISGs was maintained until 7 dpi, which might be related with gradual decrease of SARS-CoV-2 in the respiratory tract. This study establishes the importance of IFN-independent ISG activities as an innate immune inducer against SARS-CoV-2 at an early stage of infection and demonstrates that rapid induction of ISGs in the lung exerts suppressive effects against SARS-CoV-2 replication from the onset of infection. In our current study, we have not established a direct connection between the rapid upregulation of ISGs and the reduced induction of IFNs in response to SARS-CoV-2 infection. However, our RNA-seq data showed that both *Irf7* and *Irf9* mRNA levels were induced in concert with upregulated gene expression of *Stat1*, *Stat2*, *cGAS*, and *Trex1* instead of IRF3 and STING activation. While interferon plays a significant role in controlling viral infections, interferon-independent pathways for antiviral defense also exist. For example, STING can activate the transcription factor STAT6 independently of the IFN pathway, which involves the direct induction of a specific subset of ISGs by IRF3 [18]. Therefore, additional research will be necessary to prove various transcriptional mechanisms of IFN-independent ISG induction in SARS-CoV-2-infected lungs.

Our present study offers fundamental information regarding the rapid induction of innate immune response provoked by SARS-CoV-2 infection and the detailed description of IFN-independent innate immune responses in the lung of an *in vivo* model. However, our study only examined the transcription of antiviral genes and did not show protein expression or functional activity. Whether such IFN-independent activation of immune responses will require to be proved via protein data, and major findings of innate immune responses in COVID-19 patients will be added to differentiate favorable outcomes about rapid induction of innate immune responses against SARS-CoV-2.

The present data show that the transcription of ISGs is rapidly induced in the lung of SARS-CoV-2-infected hamsters after the onset of viral replication, irrespective of IFN signaling. Rapid ISG upregulation in the lungs of hamsters might be useful to restrict the transmission of SARS-CoV-2 in the early stages of infection. Our work has provided further insight into an alternative mechanism associated with IFN-independent transcription of ISGs in an *in vivo* lung to defend against SARS-CoV-2.

## 5 Declaration

### Funding statement

This work was supported by the Basic Science Research Program through the National Research Foundation of Korea, funded by the Ministry of Education (2022R1A2C2011867 awarded to HJK). This research was also supported by a grant from the Korean Health Technology R&D Project through the Korean Health Industry Development Institute, funded by the Ministry of Health & Welfare of the Republic of Korea (HI23C0795) and Ministry of Science & ICT (RS-2023-00222762), awarded to HJK.

### 5.1 Competing interest statement

The authors declare that the research was conducted in the absence of any commercial or financial relationships that could be construed as potential conflicts of interest.

### 5.2 Data availability

All data collected in this study are available from the corresponding authors upon reasonable request.

## CRediT authorship contribution statement

**Sung-Dong Cho:** Conceptualization, Data curation. **Haeun Shin:** Methodology. **Sujin Kim:** Data curation, Formal analysis. **Hyun Jik Kim:** Conceptualization, Writing - original draft.

## Declaration of competing interest

The authors declare that they have no known competing financial interests or personal relationships that could have appeared to influence the work reported in this paper.
